# Sex education for adolescent and young adult university students, a new challenge in higher education: a systematic review

**DOI:** 10.3389/fpubh.2026.1759427

**Published:** 2026-02-20

**Authors:** Carelys Montenegro-Rivera, Judith Martínez-Royert, Jordis Hernández-Martínez

**Affiliations:** 1 Universidad Simón Bolívar, Facultad de Ciencias de la Salud, Centro de Investigación en Ciencias de la Vida, Programa de Enfermería, Barranquilla, Colombia; 2 Universidad Rey Juan Carlos (URJC), Alcorcón, Spain; 3 Universidad Simón Bolívar, Facultad de Ciencias de la Salud, Programa de Enfermería, Barranquilla, Colombia

**Keywords:** adolescents, sex education, sexuality, systematic review, universities

## Abstract

**Introduction:**

Sexuality is a primordial aspect of the human being whose meaning goes beyond sex, encompassing aspects such as gender, identity, sexual preference, sensuality, affection, love and procreation.

**Objective:**

To analyze the state of knowledge, beliefs and attitudes that university students have about sexuality.

**Methodology:**

Bibliographic review of studies developed in 11 countries in English and Spanish databases: LILACS, Scopus, Dialnet, Pubmed, Researchgate, Redalycs, ScienceDirect, Proquest and SciELO; published between 2015–2025.

**Results:**

The review reveals a disconnect between students' theoretical knowledge of sexuality and its application, which facilitates the acquisition of risky behaviors. The analysis highlights that, particularly in Latin America, biological and risk-centered educational approaches continue to predominate, omitting the emotional and social dimensions in the educational sphere. In addition, gaps were identified in teacher training and in the application of effective, long-term intervention models in the university context.

**Conclusions:**

There are a large number of studies that determine low knowledge on the part of university students on issues related to their sexuality, directly influencing the practices, attitudes and beliefs that they possess. Similarly, greater support is needed from higher education institutions in the design, implementation, and monitoring of sexual and reproductive education programs, contributing significantly to the construction of a life project and reducing social inequality resulting from school dropout caused by situations such as unwanted pregnancies, sexually transmitted diseases, and gender-based violence.

## Introduction

Human beings, considered as holistic beings who develop within a social and cultural context, go through phases that determine their abilities and opportunities for development. Their wellbeing is related to the possibilities they have to develop their potential and overcome the restrictions imposed by their environment. From a humanistic perspective, their evolution is understood as an active and comprehensive process focused on self-realization and the creation of a full and dignified life (1). Experts define sexuality as a fundamental aspect of human beings whose meaning goes beyond sex, encompassing aspects such as gender, identity, sexual preference, sensuality, affection, love, and procreation; all of which manifest themselves as thoughts, desires, hopes, attitudes, and experiences, as well as being the product of the reciprocal action of biological, psychological, sociocultural, and spiritual components (2, 3). Adolescence, as the most complex and transcendental stage in the human life cycle, has become a topic of increasing interest in academic discourse and among the general population (4–6). Adolescence is the period in which human beings are not only defining their identity and personality, but also strengthening their cognitive and social dimensions (7). According to the WHO, adolescence lasts until the age of 19; however, studies propose emerging adulthood as a new developmental concept for the period spanning late adolescence and the twenties, with a particular focus on the ages of 18 to 25, a period in which human beings are at a crucial stage of consolidating their identity and sexual habits, making it a critical scenario for the materialization of the shortcomings of previous school sex education (8). In Colombia today, of the 51,609,000 inhabitants, 17% are between the ages of 10 and 19 (9), and approximately 28% are enrolled in higher education programs (2,448,271) (10), In Colombia, approximately 10% of people aged 15 to 19 were enrolled in tertiary programs (higher education) in 2021, while the rest were in secondary (high school) or other levels (11), which represents a challenge for educational institutions in providing comprehensive training for these adolescents given their youth, inexperience, and, above all, because this is a stage in which behaviors and patterns of behavior are modeled for the stages of the cycle to come. Sex education plays a leading role in this challenge (12–16). Young people engage in more risky and dangerous behaviors than the adult population (17, 18); at this stage of life, sexual maturity is achieved before emotional maturity.

Most young people reach sexual maturity much earlier than in other dimensions (social, cognitive, emotional), which is usually linked to an active sex life at an early age characterized by a lack of use of methods to prevent pregnancy and frequent changes of sexual partners (19).

Over time, there has been much debate about the relevance of sex education depending on the context in which the individual is immersed. Some argue that it is necessary to educate children about sexuality from an early age and to continue this education into adulthood. Similarly, there have been many debates among those who question the teaching of sex education to adolescents, considering it unsuitable for their mental and social age or believing that those involved in the teaching process require further training and commitment (17, 18). In the Colombian context, sex education has been replicated for generations without real questioning, limiting itself to the transmission of content without addressing sexuality in the classroom, which has fragmented educational intervention by omitting ethical, physiological, and emotional aspects (19). Colombia currently has the Program for Sexuality Education and Citizenship Building (PESCC) (20) which aims to promote a rights-based approach and identity building. However, there is still a disconnect between this and other public policies and the reality of classrooms, with sexuality being addressed in a superficial manner or limited to the biological (21).

The issue of sexuality among young people has resonated and is of global interest, so that addressing it from and toward their education is presented as an emerging challenge, from which not only responsible sexuality is ensured but also the development of awareness of the social problems linked to this constitutive dimension of human development, subject not only to the biological and physical, but also to the psychological, the cultural and historical context, and ethical principles (22), which involve emotions, behaviors, thought processes, and communication (23); it is a comprehensive, personalizing, and humanizing concept.

University education has focused on training professionals capable of exercising or executing skills inherent to their career or profession, that is, people who are competent according to the needs of the world of work and the context. To this end, teachers have developed pedagogical strategies for the development of these skills and abilities. However, the individual‘s attitude toward the apprehension and application of this new knowledge is fundamental for an effective and comprehensive education. Similarly, the approach taken from education on the dimensions that make up the human being, from a holistic perspective, to strengthen young people's ability to cope with real situations that directly affect their development, is part of this great educational challenge. The practice of sexuality has consequences for the individual; it plays an important role in their self-image and in the acceptance of others (24), considering that poorly managed sexuality can result in unwanted pregnancies, sexually transmitted diseases, among other aspects, which would directly affect their academic development, reflected in total or partial abandonment of studies, decreased job opportunities, and a low probability of improving their quality of life in the future (24).

The lack of approach and guidance on the part of teachers, the precarious or non-existent transversality of sex education, as well as recurrent isolated educational interventions due to the lack of implementation of intervention programs related to the subject, has made it difficult to achieve the expected results in sex education. revealing the urgent need for training students to contribute to responsible sexual practices (25, 26).

There is an urgent need to educate human beings capable of taking a critical stance toward today's globalizing society and to be able to contextualize everyday situations in the classroom in order to generate collective knowledge. Similarly, it has become an educational challenge to structure effective teaching methods that reinforce independence or emancipation and encourage decision-making (25, 27), awakening the interest of teachers and students in constantly learning about, recognizing, signifying, and re-signifying the realities and concepts that arise from each area of knowledge. Sexuality is a real process, inherent to the human condition, which is not far removed from everyday life, even more so in the university setting, where, in addition to their professional training, young people are tirelessly searching for elements that will shape their own personality and character with which they will face the productive or occupational context.

Educational organizations recognize the importance of comprehensive education with actions aimed at sex education from a cross-curricular approach and with psychological support and counseling. However, multiple studies indicate that, despite educational and political efforts, students continue to face significant gaps in their sex education, exposing them to risky behaviors that have a significant social and economic impact (25, 26, 28). This is reflected in fewer educational and employment opportunities, poor academic performance, and partial or total school dropout, perpetuating the cycle of exclusion and inequality (29–31).

This article therefore proposes a review and summary of the main findings of research related to the conceptualization of sexuality, the perception of sexuality among adolescents and teachers, and the vision and mission proposed by educational organizations for addressing sexuality among their student populations.

## Materials and methods

An integrative literature review was conducted using different systematization elements, comparing and analyzing various scientific publications from eleven countries (Spain, Canada, Mexico, Chile, the United States, Brazil, Peru, Costa Rica, Cuba, Ecuador, and Colombia) from 2015 to 2025. To ensure a comprehensive review, the search strategy included scientific journals, government reports, theses, and documents from international organizations such as the WHO and UNFPA. When selecting the databases for the information search, it was taken into account that adolescent sexuality has been addressed from various perspectives and disciplines (psychological, religious, philosophical), especially by branches of health (nursing, medicine) and education. Therefore, eight databases specializing in these areas were used, including publications from different regions (LILACS, Scopus, Dialnet, PubMed, Researchgate, Redalycs, ScienceDirect, Proquest, and SciELO), using search equations in both Spanish and English (Table 1).

**Table 1:** Search equations.

**Database**	**Search equation**
LILACS	(tw:(EDUCACION)) AND (tw:(SEXUALIDAD)) (tw:(SEXUALIDAD)) AND (tw:(UNIVERSITARIOS)) (tw:(EDUCACION)) (SEXUAL)) AND (tw:(EDUCACION SUPERIOR))
Redalycs	Sexualidad “y” adolescencia Educación sexual “o” sexualidad
SciELO	Educación sexual AND adolescents Sexualidad AND Universitarios
Dialnet	Sexualidad “y” Adolescencia Educación “y” Sexualidad Sexualidad “y” Universidad
Reserchgate	Sexualidad Educación sexual en adolescentes
PUBMED	(sexuality) AND teenagers (sex education) AND university
Scopus	(Sexuality AND university/AND teenagers OR Students) AND LIMIT-TO (PUBYEAR, 2015) OR LIMIT-TO (PUBYEAR, 2015–2025) OR LIMIT-TO (PUBYEAR, 2015–2025)
ScienceDirect	SEXUALITY and STUDENTS. (SEX EDUCATION) and UNIVERSITY.

Source: Prepared by the author.

Initially, no language restrictions were applied during the search phase; however, the final selection only included studies in English, Spanish, and Portuguese due to the availability of complete information, which were published within the defined time frame and addressed sexuality from any of the dimensions of human development. The target population was defined as adolescents, late adolescents, and emerging adults (aged 18 to 25) enrolled in higher education programs. This age range was defined in order to cover the transition period between school and university life. Editorials and publications that addressed sexuality from a purely biological perspective (contraception, human papillomavirus/HIV, among others) without an academic context were discarded. Thus, 142 publications were excluded, and 52 articles that met the variables of interest were selected.

**Figure 1:**
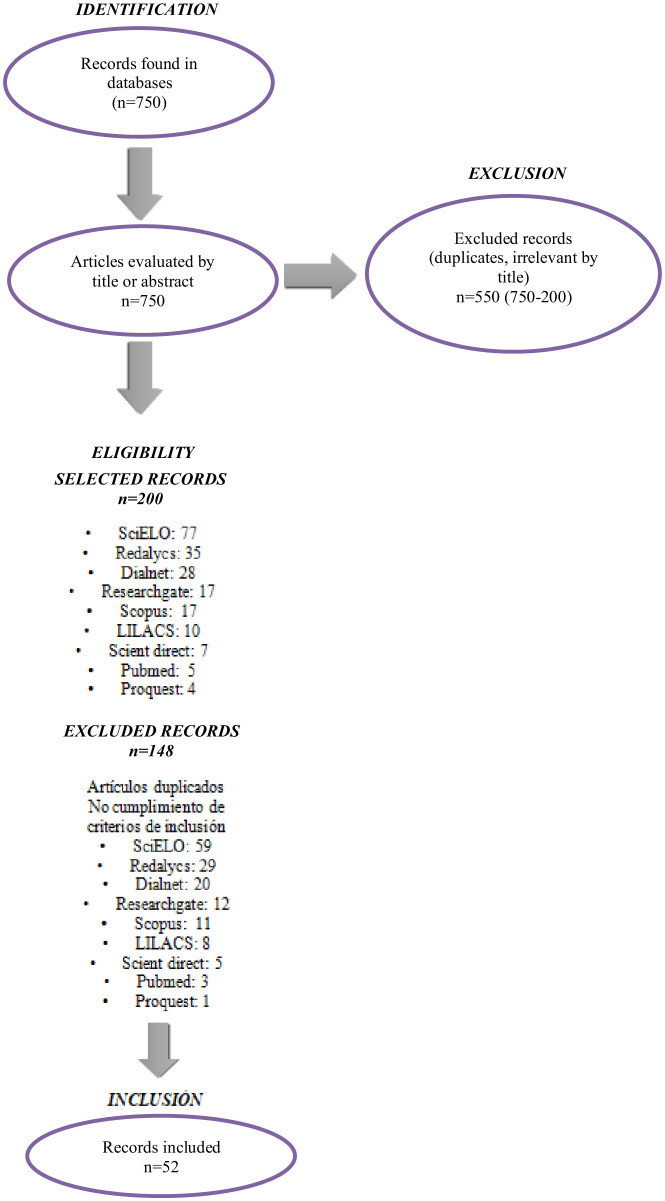
Flowchart of article selection based on the reviewed literature. Source: Prepared by the author.

For data analysis, a specially designed “Literature Review Form” was created, defined in three dimensions. In the first phase, identification information was entered: title, author, country, year of publication, journal, database, search equation, results, and main conclusions, which were classified into: Research articles, which allowed for a review of the results produced by a research process; Review articles, which facilitated the understanding and conceptualization of sexuality and the identification of theoretical references; and articles excluded according to the established criteria. In the second phase, methodological aspects such as type of study and target population were taken into account. The third phase considered aspects related to the central theme and its findings (knowledge, attitudes, barriers for teachers, public policies, and their analysis; Figure 1).

## Results

The analysis of the articles that met the selection criteria facilitates the categorization of the results from three dimensions: degree of knowledge and practices, differences and similarities from different geographical contexts, and gaps in sexuality research from this perspective.

The assumptions and results of the studies shows that there is a prevalence of early sexual initiation without the necessary knowledge (32, 33), which leads to risky behaviors and student dropout rates. Similarly, topics related to sex education are rarely discussed by teachers in areas other than health, religion, or biology (34), and there is little training for teachers in sex education strategies and tools for identifying risky sexual behavior in middle school and high school students (35–37). With regard to knowledge and practices, the review reveals a consensus in the literature on the disconnect between theory and practice. Although the results of the studies show that university students have an acceptable theoretical level of knowledge on topics such as pregnancy prevention and STIs, this does not translate into preventive behaviors (condom use). Recent studies of adolescent and young university populations in Latin America and Europe show that condom use remains low, despite access to information (38).

In the Latin American context (Colombia, Chile, Mexico), a “biological” and “risk-based” approach continues to predominate, focused almost exclusively on the prevention of disease and unplanned pregnancies, generating myths and taboos around issues such as pleasure, diversity, and emotional relationships (14, 39). Countries such as Cuba have made some progress in teacher training on issues related to sexual health for trainers of trainers through pedagogical strategies focused on sex education (40). Although this is the situation in Latin America, reviews that include programs in Spain and Portugal, on the contrary, show greater integration of Comprehensive Sexuality Education (CSE) into formal education, with a focus on gender perspective. However, it is observed that in both contexts the challenge remains of counteracting the excess of information from “informal sources” (the internet, peers, pornography), which tend to have a greater impact on young people's sexual attitudes than the formal education they receive (41, 42).

In conclusion, there are still significant gaps in research that limit a comprehensive understanding of the phenomenon, as well as a lack of evaluation of the long-term effectiveness of educational interventions in modifying risky sexual behaviors (43).

There is a lack of attention to issues such as gender identity and sexual orientation that would allow for an analysis of sex education from this perspective (33).

Much of the literature continues to focus on women's reproductive responsibility, with few studies examining the role of men and their responsibility in sexual relationships. Finally, the analysis leads to the conclusion that studies are needed on the theoretical and pedagogical training of university teachers to address sexuality beyond the biological (40). Teachers have a notable difficulty in talking about sexuality with students, mainly due to their lack of knowledge about sexuality, as well as their experience of it as a taboo, prohibition, fear, and guilt, avoiding confrontation due to the distress it causes them (44–46).

## Discussion

Statistics indicate that unwanted pregnancy remains a significant problem in many countries. According to the WHO, there are approximately 121 million unwanted pregnancies worldwide each year. Nearly half of all pregnancies worldwide, a total of 121 million, are unintended (47). Unwanted pregnancy rates are higher in countries with limited access to contraception and sex education, as well as in marginalized and disadvantaged communities. Latin America and the Caribbean (LAC) are considered subregions with the second highest rate of teenage pregnancies in the world. The global rate of teenage pregnancy is estimated at 46 births per 1,000 girls, while teenage pregnancy rates in LAC remain the second highest in the world, estimated at 66.5 births per 1,000 girls aged 15 to 19 (48).

The current statistics on STIs and unwanted pregnancies worldwide are due, among other reasons, to the inadequate approach to sexuality. This situation has led to an analysis of its causal factors, highlighting the limited appropriation and application of knowledge about sexuality, the limited use of teaching aids in sex education, the poor implementation of comprehensive policies and programs for identifying and addressing risky sexual behavior, and the insufficient monitoring of this issue by school organizations (42).

The studies consulted measured not only knowledge, but also its consistency with the sexual behaviors of young people, as well as the effectiveness of various strategies, programs, or interventions that have been proposed by some educational organizations focused on mitigating this problem. each of which has made significant contributions to a greater or lesser extent in terms of knowledge acquisition, identification of barrier and/or prevention tools, and guidance for comprehensive care for risky sexual behaviors. However, there is a clear need for an interdisciplinary, interinstitutional, and intersectoral approach. This coordination would facilitate the strengthening of pedagogical and educational strategies used for sexuality education in the educational sphere. It is not enough to impart knowledge; to be effective, education must make use of appropriate and effective channels that enable the modification of risky sexual behaviors (49–51).

It should be acknowledged that significant progress has been made in addressing this issue in terms of diagnosing the causes and consequences of its inadequate management. To date, the scope and limitations of these interventions remain unclear, a situation that has been confirmed by some studies which assert that programs aimed at strengthening sex education are deficient or non-existent in the university setting; furthermore, they assert that families and universities do not contribute to the education of adolescents (52, 53).

Education is a key component in promoting responsible sexuality (49), beginning before the onset of sexual relations and continuing through university education, thereby strengthening adolescents' knowledge and application of this knowledge.

Sexuality is a key component of personal balance, and ensuring it in a safe manner becomes a basic social objective (54–56), not only in secondary and university education but also in public health policies, which should serve as a reference for educational institutions to implement programs and projects for their students (57).

On the other hand, it is necessary to delve into the limitations that adolescents face when applying their knowledge to their sexual practices, since, although they have prior information on sexual health and other related topics, they do not behave accordingly due to gaps in specific aspects that are crucial for the management of safe sexuality (57).

One limitation of this review was the heterogeneity of the studies found, which made a comprehensive statistical meta-analysis difficult and led to a descriptive interpretation of the results.

## Conclusions

The results presented in this review show researchers' efforts to address the current state of sex education among college-aged adolescents, taking into account the importance of this stage in preventing risky sexual behavior resulting from exposure to external influences that affect their personal and professional development. However, there are different strategies for addressing sexuality in adolescents, including educational processes and those that involve a variety of actors in society. With regard to the first point, greater monitoring and support is required within educational institutions, given that the characteristics of their populations vary considerably and the acceptance and application of the knowledge acquired about sexuality are closely related to the cultural patterns of each region.

Currently, a large percentage of university students are sexually active, which indicates a significant possibility of contracting STIs. Greater government commitment and the establishment of clear and measurable indicators for interventions aimed at teacher training and university students are necessary, as is continuing to generate research that contributes significantly to the design, implementation, and monitoring of programs aimed at reducing inappropriate sexuality in this population and that are coordinated with primary and secondary education, promoting the construction of a solid vision, life expectancy and the reduction of aspects such as social inequality. This is crucial considering that, at present, the impact of situations such as early pregnancy leads to school dropout and future failure in relationships, which perpetuates the cycle of poverty.
