# Correction: Sex education for adolescent and young adult university students, a new challenge in higher education: a systematic review

**DOI:** 10.3389/fpubh.2026.1925785

**Published:** 2026-07-27

**Authors:** Carelys Montenegro-Rivera, Judith Martínez-Royert, Jordis Hernández-Martínez

**Affiliations:** 1 Universidad Simón Bolívar, Facultad de Ciencias de la Salud, Centro de Investigación en Ciencias de la Vida, Programa de Enfermería, Barranquilla, Colombia; 2 Universidad Rey Juan Carlos (URJC), Alcorcón, Spain; 3 Universidad Simón Bolívar, Facultad de Ciencias de la Salud, Programa de Enfermería, Barranquilla, Colombia

**Keywords:** adolescents, sex education, sexuality, systematic review, universities

Affiliation “Universidad Rey Juan Carlos (URJC), Alcorcón, Spain” was omitted for author Carelys Montenegro-Rivera. This affiliation has now been added for author Carelys Montenegro-Rivera.

The original version of this article has been updated.

